# Acute service and disability service providers experiences of joint working to improve health care experiences of people with an intellectual disability compared to non-joint working: A mixed-method systematic review

**DOI:** 10.1177/17446295231209345

**Published:** 2023-10-21

**Authors:** Eileen Kelleher, Anne-Marie Martin, Maria Caples, Teresa Wills

**Affiliations:** 8795 University College Cork, Ireland

**Keywords:** intellectual disability, disability service, acute service, joint working, systematic review

## Abstract

Persons with intellectual disabilities require frequent access to acute services. Many also access disability services within the community. Reports and enquiries have highlighted the sub-optimal healthcare provided to this group when accessing healthcare in acute services. Joint working between acute and disability services has been identified as a measure to improve healthcare for this group. A mixed method systematic review was undertaken to explore current evidence of joint working between both service providers. Twelve publications were included, and the data were analysed using thematic analysis. Confusion around responsibility and limited training in acute services prevented joint working from occurring. Information-sharing is pivotal in promoting joint-working, but measures which facilitated it were not always used. Albeit acute services demonstrated a strong commitment to deliver quality care to those with intellectual disabilities. Much of the available research captures the experiences of staff in acute services. There is a paucity of research available exploring experiences of disability service providers.

## Introduction

Those with intellectual disabilities experience more health needs compared to those without (St. John et al., 2018) and are two times more likely to access acute services compared to the general population (McCormick et al., 2021; Glover et al., 2019; Read & Rushton, 2013). Acute services are considered providers of health care within a general hospital setting. People with intellectual disabilities are often linked to disability services which are community-based services that support people with intellectual disabilities and provide specialist support through multi-disciplinary therapies, behaviour support, residential supports, day services, and respite (Health Service Executive, 2023; Casey et al., 2020). There has been an increase in the utilization of disability services over recent years (Doyle et al., 2020). Current figures indicate that 23,763 people with intellectual disabilities are in receipt of public funded disability services in Ireland (Casey et al. 2020). A rise to that figure is expected over coming years as demand grows due to increased longevity (Emerson et al., 2014) and higher rates of complex health needs amongst this population group (Cooper et al., 2015; Cooper et al., 2017). As persons with intellectual disabilities require access to acute and disability services, joint working between both services is crucial to providing optimum healthcare (Cantrell et al., 2020).

Over the last two decades, healthcare provided to persons with an intellectual disability in acute services has received significant attention. In 2007, the report ‘Death by Indifference’ (Mencap, 2007) highlighted the lack of appropriate healthcare provided to some adults with an intellectual disability while attending acute services and ultimately contributed to premature deaths. This initiated the inquiry “Healthcare for All: The independent inquiry into Access to Healthcare for People with Learning Disabilities” (Michael, 2008) which revealed that those with intellectual disabilities experience unnecessary suffering while availing of healthcare in acute services and that many deaths are avoidable. In 2013 the ‘Confidential Inquiry into the deaths of people with learning disabilities’ determined that delayed diagnosis and treatment, poor healthcare planning and poor communication contributed to the premature deaths of 42% of the 238 deaths investigated (Heslop et al., 2013). More recently, Heslop et al. (2021) concluded that these issues remain problematic, raising continued questions over the healthcare provided to individuals with an intellectual disability while accessing acute services.

There have been both international and national responses to address the inadequate healthcare provided to those with intellectual disabilities in acute services. The United Nations Convention on the Rights of Persons with Disabilities (UNCRPD) was introduced to promote and protect the rights of all persons living with a disability (United Nations, 2006). Article 25 declares that all persons with a disability have equal entitlement to the same quality and range of healthcare as the general population. The UNCRPD has been a driving force for change in the provision of healthcare for those with disabilities and is deeply rooted in the development and implementation of programmes, policies, and legislation nationally and internationally.

The European Declaration on the Health of Children and Young People with Intellectual Disabilities and their Families: ‘Better Health, Better Lives’, (WHO, 2010) sets out research and policy priorities to address inequalities encountered by persons with intellectual disabilities. Priority 5 is concerned with the need for quality health services for those with intellectual disabilities and indicates that joint working between services is one measure in achieving this (WHO, 2010). Ten years on from ‘Better Health, Better Lives’ the need for joint working and coordinated healthcare between services are reiterated to improve the delivery of healthcare for persons with intellectual disabilities (Scherer et al., 2022).

On a national level, the UNCRPD has underpinned several policies in Ireland to improve healthcare of persons with an intellectual disability. In 2011, Ireland saw the introduction of the policy ‘Time to Move on From Congregated Settings’ (Health Service Executive, 2011) which details the transition from large, congregated settings to smaller community-based housing for persons living with an intellectual disability. The transition to community implies accessing the same services as available to the general population (Health Service Executive, 2011). In the past, care of people with intellectual disabilities who were ill was often managed by general practitioners (GP’s) and nurses on site in intellectual disability services. Nowadays with a move to the community, those with intellectual disabilities are now accessing primary care services, where referrals to acute services may be more frequent, therefore placing a further demand on services.

As health systems strive to improve healthcare for individuals with an intellectual disability in acute services, numerous measures have been recommended to achieve this. Such measures have included the introduction of intellectual disability liaison nurse post in acute services and the use of hospital passports (McCormick et al., 2021). Joint working between acute and disability service providers has also repeatedly been identified as one such measure (Moloney et al. 2021; Cantrell et al., 2020; WHO, 2010). Joint working, also referred to as collaboration, partnership, inter-agency or multi-agency working, involves two or more organisations working together to achieve a common goal (Alderwick et al., 2021). The concept of joint working is to improve the efficiency of services involved, prevent duplication of work, and improve planning and co-ordination of care for patients (Crocker at al., 2020). McConkey (2005) indicates that an absence of joint working between services, can result in a poorer standard of healthcare for those with intellectual disabilities. Lewis and Stenfert-Kroese, (2010) concluded that further exploration of joint working between acute and disability service providers is needed to understand its impact. A public consultation on the ‘Disability Action Plan 2022 – 2025’ agreed that more focus and commitment is required on joint-working between acute and disability service providers to improve healthcare for those with disabilities (Department of Health, 2021).

### Aim

As joint working between acute and disability service providers has been identified as a measure to improve healthcare for people with intellectual disabilities in acute services, the aim of this systematic review was to examine current evidence of acute and disability service providers experiences of joint working to improve healthcare of people with intellectual disability compared to non-joint working. More specifically the review sought to answer the following questions:

(1a) What are acute service providers experiences of joint working with disability service providers?
(1b) What are acute service providers experiences of non-joint working with disability service providers?
(2a) What are disability service providers experiences of joint working with acute service providers?
(2b) What are disability service providers experiences of non-joint working with acute service providers?

## Methods

### Study design

A mixed method systematic review (MMSR) approach was used as the current body of evidence includes studies using different research designs (Lizarondo et al., 2020). This MMSR was conducted in line with the Preferred Reporting Items for Systematic Reviews and Meta-analysis (PRISMA) guidelines. A research protocol was developed and registered on PROSPERO (June 2022). Database searches of SCOPUS and EBSCO were carried out and no existing MMSR on this topic was identified.

### Search strategy

Searches in two databases, EBSCO and SCOPUS were conducted in May 2022. EBSCO was used to search CINAHL, MEDLINE and Academic Search Complete databases. Collectively these databases offer literature extending across a broad range of research areas pertinent to intellectual disability in areas such as nursing, medical and allied health journals. Key concepts of the search included 1. “acute service*” OR “acute setting*” OR “tertiary care” OR hospital* 2. Joint-working OR partners* OR collab* OR integrat* OR inter-agency OR multi-agency OR liaison 3. “Intellectual disabilit*” OR “Mental retard*” OR “learning disabilit*” OR “learning difficult*” OR “Developmental disabilit*” 4. service* OR “service provider” OR organization (Table 1). Studies were limited to peer-reviewed journals, English language publications, and publication date within the last 16 years (since 2006) to account for studies post the enactment of the UNCRPD and subsequently reflect changes in policy. The results from each database search were imported to Rayyan (Ouzzani et al., 2016), to assist in the organisation and management of collaborative systematic reviews.

**Table 1.** Databases search strategy.

EBSCO Search		
Concept	Key Headings	Results
S1.	TI (“acute service*” OR “acute setting*” OR “tertiary care” OR hospital*) OR AB (“acute service*” OR “acute setting*” OR “tertiary care” OR hospital*)	2,857,661
S2.	TX Joint-working OR partners* OR collab* OR integrat* OR inter-agency OR multi-agency OR liaison	7,257,405
S3.	TI (“Intellectual disabilit*” OR “Mental retard*” OR “learning disabilit*” OR “learning difficult*” OR “Developmental disabilit*”) OR AB (“Intellectual disabilit*” OR “Mental retard*” OR “learning disabilit*” OR “learning difficult*” OR “Developmental disabilit*”)	149,193
S4.	TX service* OR “service provider” OR organization*	13,022,149
	S1 AND S2 AND S3 AND S4	843
	Limiters applied: Date (2006-2022) Published in English Academic Journals	509
SCOPUS SEARCH		
CONCEPT	KEY HEADINGS	RESULTS
S1.	TI (“acute service*” OR “acute setting*” OR “tertiary care” OR hospital*) OR AB (“acute service*” OR “acute setting*” OR “tertiary care” OR hospital*)	2,606,989
S2.	TX Joint-working OR partners* OR collab* OR integrat* OR inter-agency OR multi-agency OR liaison	12,741,829
S3.	TI (“Intellectual disabilit*” OR “Mental retard*” OR “learning disabilit*” OR “learning difficult*” OR “Developmental disabilit*”) OR AB (“Intellectual disabilit*” OR “Mental retard*” OR “learning disabilit*” OR “learning difficult*” OR “Developmental disabilit*”)	149,473
S4.	TX service* OR “service provider” OR organization*	16,290,820
	S1 AND S2 AND S3 AND S4	1016
	Limiters applied: Date (2006-2022) Published in English Academic Journals	685

## Study selection

### Inclusion criteria

1. Healthcare professionals /paid carers (such as support workers, personals aids) working in disability services that supported people with intellectual disabilities within acute services.
2. Healthcare professionals/paid carers (such as support workers, personals aids) working in acute services that supported people with intellectual disabilities within acute services.
3. Primary research papers.

### Exclusion criteria

1. Healthcare professionals/paid carers working in disability that have not supported people with intellectual disabilities within acute services.
2. Healthcare professionals/paid carers working in acute services that have not supported people with intellectual disabilities within acute services.
3. Healthcare professionals/paid carers working in all other health care settings were excluded (acute mental health services, palliative care, primary care).
4. Secondary research papers (systematic review, literature review, discussion papers, commentary papers, conference papers, abstracts, and posters).

### Data extraction

Data extraction was undertaken by the first author (EK) and reviewed by the other three authors (AMM,MC,TW). The data extracted included author, year, country, research question/aim, research design, sample, setting, data collection methods, data analysis, key findings (Table 3).

**Table 2.** PRISMA Flow Diagram.

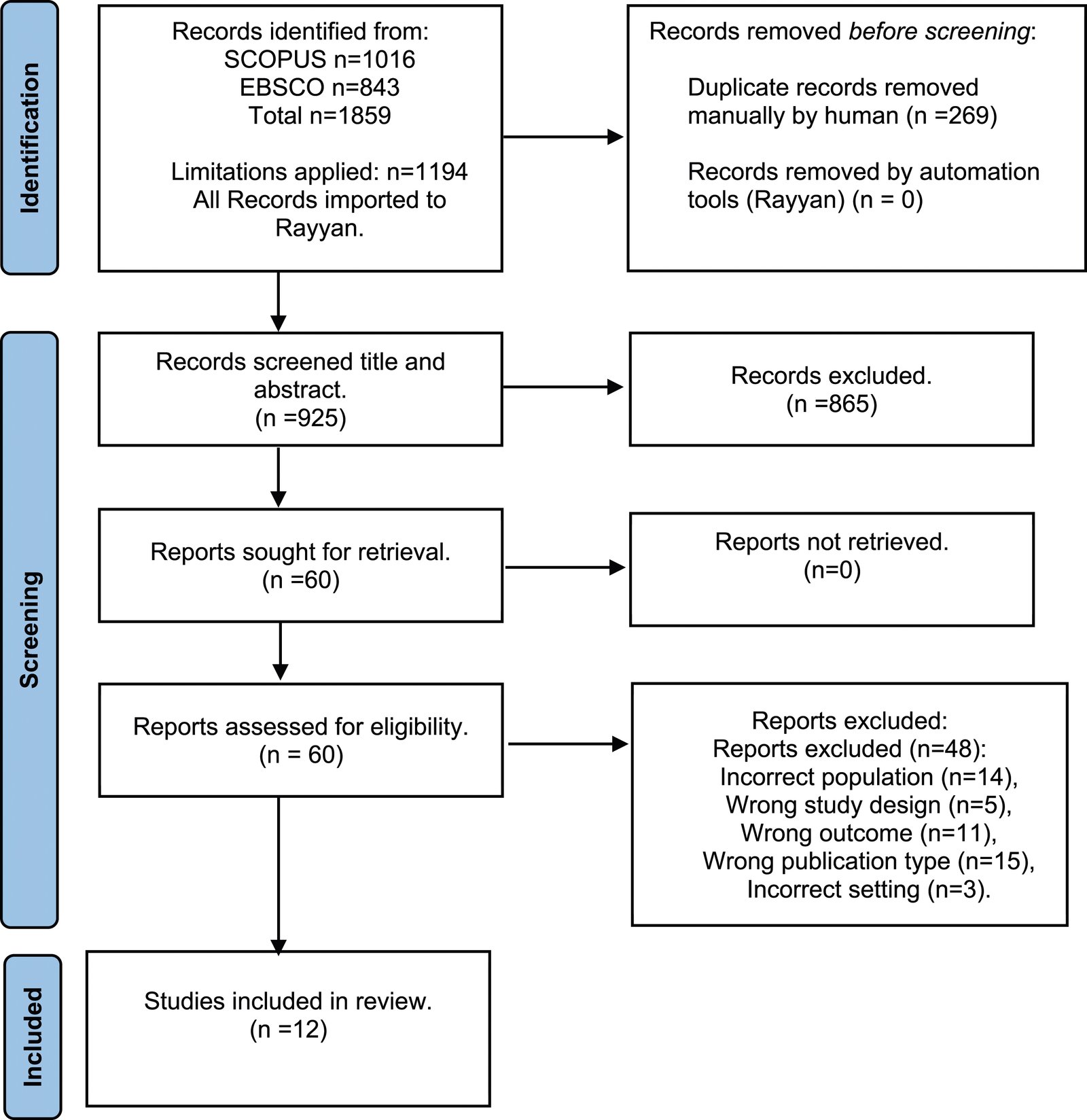

**Table 3.** Data Extraction tables.

Author Year Country	Research question Or study aim	Research design	Sample/ setting	Data collection methods	Data Analysis	Key findings
Oulton et al. (2018) United Kingdom	Describe organisational context for healthcare delivery and compare staff perceptions of their ability to identify the needs of children and young persons (CYP) with/without an intellectual disability and their families.	Mixed method	Children’s hospitals and non-children’s hospital. Staff in a senior clinical or managerial role (n=65) Clinical and non-clinical staff (n=2261)	semi-structured interviews and anonymised Surveys	Thematic analysis Descriptive statistics	Few or no policies, systems, and practices for people with intellectual disabilities in hospitals. Hospitals felt incapable of caring for those with intellectual disabilities, lack of knowledge. Flagging and identifying systems in place. Lack of communication between organisation, staff, and family. Hospital passports beneficial but no organisational support. Specific intellectual disability training enables staff to access specialist resources when required. Poor coordination between hospital services.
Oulton et al. (2019) United Kingdom	Identify extent of dedicated intellectual disability nurse provision in children’s hospitals and compare perceptions of staff working in hospitals with intellectual disability liaison nurse provision compared to those working in hospitals without.	Mixed methods	Children’s Hospital & non-children’s hospitals. Senior hospital staff (n=48), Clinical and non-clinical staff (n=1681)	Semi-structured interviews. Anonymous survey.	Framework approach. Descriptive statistics.	Specialized intellectual disability team in hospitals contribute to better care. Hospitals described as *‘far from perfect’* but committed to improving care. Emergency admissions challenging for staff. Identifying patients with intellectual disabilities crucial. Good practice embedded over time. Training for hospital staff crucial. Role of LD nurse include collaborating and developing systems, pathways and policies and educating staff.
MacArthur et al. (2015) Edinburgh, United Kingdom	Examine the role of the intellectual disability liaison nurse in facilitating reasonable and achievable adjustments for persons with intellectual disabilities.	Mixed method	liaison nurse (n=6) Adults with mild/moderate intellectual disabilities (n=5), family or paid carers (n=16), primary health care professionals from all disciplines (n=39), secondary healthcare professionals (n=19)	Referral data collected over 18-month period, Semi-structured interviews & focus groups		Liaison nurse in acute services responsible for co-ordinating care, implementation reasonable adjustments, support and prepare hospital staff, flagging, implements hospital passport.
Bell (2012) United Kingdom	To evaluate a hospital passport tool designed to improve communication between people with intellectual disabilities and hospital staff.	Qualitative	Health, hospital, and social care workers (n=20), Family carers (n=12), Persons with intellectual disabilities (n=8)	Focus groups & semi-structured interviews	Reflexive journal analysis	Hospital passport supports communication between all agencies. Poor communication identified issue. Limited knowledge/training linked to reduced confidence in hospital staff. Community staff supporting patients with intellectual disability unfamiliar with their needs. Hospital system fail to support multi-agency working. Clear pathways and information systems required to support patients with intellectual disabilities. At times hospitals lack commitment to provide adequate care for patients with intellectual disabilities.
Drodz and Clinch (2016) United Kingdom	Explore the experiences of orthopaedic and trauma nurses who have cared for persons with an intellectual disability.	Mixed methods	Registered orthopaedic and trauma nurses (n=13)	Questionnaire	Descriptive statistical analysis. Thematic analysis	Poorer care for patients with an ID compared to those without. Communication issues identified. Lack of competence in caring for those with an intellectual disability. Compassion shown to those with intellectual disabilities the same as those without. Reasonable adjustments facilitated. Lack of knowledge and understanding of intellectual disability in hospitals. Direct care delivered by the carer, not hospital staff. Promoting positive partnerships with all carers crucial. Access to liaison nurse not always available.
Brown and Guvenir (2008) United Kingdom	Investigate the experiences of children with an ID, their carers and nursing staff during a hospital admission.	Qualitative	Carers (n=13) and hospital staff (n=13).	Semi-structured interviews	Thematic analysis.	Feeling of anxiousness when caring for a child with an ID Emergency admissions cause fear in staff. Hospitals not equipped to cope with persons presenting with behaviour concerns. Carers relied upon to provide direct care.
Brown et al. (2012) United Kingdom	Examine the impact and outcomes of four intellectual disability liaison nurse services in south-east Scotland on the healthcare experiences of people with an intellectual disability attending hospitals.	Mixed methods	Adults with ID (n=5) carers (n=16), primary healthcare professionals (n=39), secondary healthcare professionals (n=19), liaison nurses (n=3)	Semi-structured interview, focus groups, collection of liaison nurse activity data.	Thematic analysis	Community nurses made most referrals to LDLN service within the hospitals. Role of the LDLN service included: Facilitation of Co-ordinating care, bridge between all services, involved in the client’s care, implementation of reasonable adjustments, training staff within hospital. LDLN service only operated Monday-Friday 9-5.
Castles (2012) United Kingdom	Evaluate persons with an intellectual disability, their carer and ward staff views of the work of intellectual disability liaison nurse in hospital.	Mixed methods	Patients with intellectual disability (n=7), parent carers (n=5), paid carers (5),care manager (n=1), hospital senior nurses (4), hospital physiotherapist (n=1), hospital discharge planner (n=1), hospital staff nurses (n=5), hospital support worker (n=1),community intellectual disability nurses (n=5), community intellectual disability support worker (n=1)	Semi-structured interviews and review of referral data over a 6 month period.	Thematic analysis	Liaison nurse bridges gap between services, contributes to better care in hospitals, facilitates discharge planning, contribute to information-sharing required. More visibility of liaison nurses needed.
Tuffrey-Wijne et al. (2014) United Kingdom	To identify the factors that promote and compromise the implementation of reasonably adjusted healthcare for patients with an intellectual disability in acute national health services (NHS) hospitals.	Mixed methods	Healthcare professionals, staff in managerial roles, adults with intellectual disabilities, carers of adults with intellectual disabilities, and expert panel members (n=1251)	Questionnaires, Interviews, discussion group	Analytic framework	Lack of appropriate flagging system. Poor understanding of intellectual disabilities in hospitals. No clear lines of responsibility or accountability for implementing reasonable adjustments. Role of the liaison nurse effective but requires managerial support. Lack of training in intellectual disability.
Oulton et al. (2015) United Kingdom	Understand the needs of children and young people with learning disabilities and their families when they are in hospital.	Ethnographic study	Hospital ward staff (n=135)	Observations of a hospital ward, informal conversations, discussions, review of ward documentation and interviews	Framework approach	Lack of in-hospital training. Identifying persons with intellectual disability in hospital challenging for staff. Increased information sharing between all agencies required.
Lewis and Stenfert-Kroese (2010) United Kingdom	Investigate the attitudes and emotional reactions of nursing staff towards patients with intellectual disabilities in hospital.	Quantitative	Hospital wards Study 1: general nurse (n=34), student nurse (n=1), nursing assistant (n=19) Study 2: general nurses (n=160), student nurse (n=16) nursing assistant (n=86)	Questionnaire	Inferential statistics. Descriptive statistics.	Hospital staff less positive about caring for persons with intellectual disabilities. Communication, training, and skills insufficient to care for those with intellectual disabilities. Nursing staff rely on carers to stay in hospital.
Spassiani et al. (2020) Canada	To investigate Emergency Department nurses knowledge, skills and comfort when caring for patients with intellectual disabilities (ID).	Quantitative	Emergency department nurses (n=15)	Questionnaire	Descriptive statistics	Few nurses had knowledge of intellectual disability Limited awareness of community resources for people with intellectual disability. Lack of appropriate flagging systems for persons with ID.

### Data transformation

This systematic review consisted of studies with qualitative (n=3), quantitative (n=2) and mixed-methods design (n=7). Therefore, data transformation was required to allow the evidence to by analysed. Quantitative data was transformed to textual descriptions which is known as qualitized data. The qualitized data was merged with qualitative data to allow data analysis to take place (Lizarondo et al., 2020). Quantitative data was qualitized as most of the data was qualitative.

### Quality assessment

The Mixed Method Appraisal Tool (MMAT) was completed to assess the methodological quality of the included studies (Table 4). It consists of two parts, the checklist, and the clarification of the criteria and can be completed for qualitative research, randomized control trials, non-randomized control trials, quantitative descriptive studies, and mixed methods studies (Hong at al., 2018). It is encouraged to determine the quality of the study based on a detailed presentation of the criteria rather than the methodological scoring, therefore no studies were excluded on this basis (Hong at al., 2018). The MMAT was completed independently by the authors.

**Table 4.** Mixed Method Appraisal Tool (MMAT) summary table.

	Screening questions for all studies		Qualitative					Quantitative randomized controlled trials					Quantitative non-randomized					Quantitative descriptive					Mixed methods				
	S1	S1	1.1	1.2	1.3	1.4	1.5	2.1	2.2	2.3	2.4	2.5	3.1	3.2	3.3	3.4	3.5	4.1	4.2	4.3	4.4	4.5	5.1	5.2	5.3	5.4	5.5
Oulton et al. (2018)	✓	✓	✓	✓	✓	✓	✓											✓	✓	✓	✓	✓	✓	✓	✓	✓	✓
MacArthur et al. (2015)	✓ Pg 1555	✓	✓ Pg 1156	✓	✓	✓	✓											✓ Pg 1555	✓	✓	✓	✓	✓	✓	✓	✓	✓
Oulton et al. (2019)	✓	✓	✓	✓	✓	✓	✓											✓	✓	✓	✓	✓	✓	✓	✓	✓	✓
Bell (2012)	✓ Pg 57	✓	✓ Semi Structured interviews, Focus groups, Reflexive journal analysis	✓	✓	✓	✓																				
Drodz & Clinch (2016)	✓ Pg 15	✓																✓ convenience sample	✓	✓	✓ 25 potential participants, 13 responses	✓					
Brown & Guvenir (2008)	✓	✓	✓ Semi structured interviews	✓	✓	✓	✓																				
Tuffrey-Wijne et al. (2014)	✓	✓	✓	✓	✓	✓	✓											✓	✓	✓	✓	✓	✓	✓	✓	✓	✓
Castles (2012)	✓ Pg 16	✓	✓	✓	✓	✓	✓											✓	✓	✓	✓	✓	✓	✓	✓	✓	✓
Brown et al. (2012)	✓ Pg 1163	✓	✓ Semi-structured Interviews Focus groups	✓	✓	✓	✓											✓ Purposeful sampling	✓	✓	✓	✓	✓	✓	✓	✓	✓
Oulton et al. (2015)	✓	✓	✓	✓	✓	✓	✓																				
Spassiani et al. (2020)	✓ Intro	✓																✓ Convenience sample	✓	✓	✓	✓					
Lewis & Stenfert-Kroese (2010)	✓ Pg 356	✓																✓	✓	✓ Pilot Study done	✓ Response rates varied	✓					
Oulton et al. (2018)	✓	✓	✓	✓	✓	✓	✓											✓	✓	✓	✓	✓	✓	✓	✓	✓	✓
MacArthur et al. (2015)	✓ Pg 1555	✓	✓ Pg 1156	✓	✓	✓	✓											✓ Pg 1555	✓	✓	✓	✓	✓	✓	✓	✓	✓
Oulton et al. (2019)	✓	✓	✓	✓	✓	✓	✓											✓	✓	✓	✓	✓	✓	✓	✓	✓	✓
Bell (2012)	✓ Pg 57	✓	✓ Semi Structured interviews, Focus groups, Reflexive journal analysis	✓	✓	✓	✓																				
Drodz & Clinch (2016)	✓ Pg 15	✓																✓ convenience sample	✓	✓	✓ 25 potential participants, 13 responses	✓					
Brown & Guvenir (2008)	✓	✓	✓ Semi structured interviews	✓	✓	✓	✓																				
Tuffrey-Wijne et al. (2014)	✓	✓	✓	✓	✓	✓	✓											✓	✓	✓	✓	✓	✓	✓	✓	✓	✓
Castles (2012)	✓ Pg 16	✓	✓	✓	✓	✓	✓											✓	✓	✓	✓	✓	✓	✓	✓	✓	✓
Brown et al. (2012)	✓ Pg 1163	✓	✓ Semi-structured Interviews Focus groups	✓	✓	✓	✓											✓ Purposeful sampling	✓	✓	✓	✓	✓	✓	✓	✓	✓
Oulton et al. (2015)	✓	✓	✓	✓	✓	✓	✓																				
Spassiani et al. (2020)	✓ Intro	✓																✓ Convenience sample	✓	✓	✓	✓					
Lewis & Stenfert-Kroese (2010)	✓ Pg 356	✓																✓	✓	✓ Pilot Study done	✓ Response rates varied	✓					

✓ Yes ✖ No O Can’t tell.

### Data analysis

Braun and Clarke’s (2006) thematic analysis approach was adopted as it presented an opportunity to gain an understanding of patterns and shared experiences amongst participants within the studies. The analysis of the data was achieved by using Braun and Clarke (2006) six-step framework. The first step involved the author becoming familiar with the data which was achieved by reading and re-reading the studies included in this systematic review. Step two required generating initial codes, which involved recognizing data within the studies that were linked. Following this, the codes were examined to search for themes and an overarching theme: *Preparedness* became evident and a further three themes became apparent *1. Responsibility to improve healthcare, 2. Information-sharing between service providers and 3. Knowledge, awareness, and training.* Step four involved reviewing and questioning the themes to access the quality of each theme before moving to step five which required the authors to define the themes and ensure they were relevant to the research question. Lastly the themes were written and are presented in the results section of this paper.

### Research integrity

This systematic review was conducted in line with the Preferred Reporting Items for Systematic reviews and Meta-Analyses (PRISMA), which assists researchers with the transparent reporting of systematic reviews (Page et al., 2021). A research protocol was developed and published on PROSPERO. Publishing a research protocol ensures that the methods, analysis, and results correspond with the authors initial objective (Dane, Klein Gebbink, & van der Kuy, 2023), therefore increasing the quality and transparency of the review (Ohtake & Childs, 2014).

## Results

A total of 1194 papers were retrieved from the database searches and imported to Rayyan. 269 duplicate papers were cross-checked and removed by the authors and 925 papers remained. Titles and abstracts were then screened independently by two reviewers. Following this, a further 865 studies were excluded based on eligibility criteria. 60 studies were retrieved for full text review. All authors undertook full text screening. A further 48 articles were removed as they involved incorrect population (n=14), wrong study design (n=5), wrong outcome (n=11), wrong publication type (n=15) and incorrect setting (n=3). Discussion amongst the research team resolved any discrepancies in eligibility. Twelve papers met the eligibility criteria (Table 2)

Of the twelve studies, seven were mixed-methods (Brown et al., 2012; Castles et al., 2012; Tuffrey-Wijne et al., 2014; McArthur et al., 2015; Drodz and Clinch, 2016; Oulton et al., 2018; Oulton et al., 2019), three were qualitative (Brown and Guvenir, 2008; Bell, 2012 & Oulton et al., 2015) and two were quantitative (Lewis and Stenfert-Kroese, 2010 & Spassiani et al., 2020). One was conducted in Canada (Spassiani et al., 2020) and all remaining studies were conducted in the United Kingdom (Brown and Guvenir, 2008; Lewis and Stenfert-Kroese, 2010; Brown et al., 2012; Bell, 2012 &; Castles et al., 2012; McArthur et al., 2015; Tuffrey-Wijne et al., 2014; Oulton et al., 2015; Drodz and Clinch, 2016; Oulton et al., 2018; Oulton et al., 2019).

All studies included the experiences of healthcare professionals in acute services. Only six studies consider the experiences of healthcare professionals in the community (Brown and Guvneir, 2008; Brown et al., 2012; Bell, 2012; Castles, 2012; Tuffrey-Wijne et al., 2014; MacArthur et al., 2015). No study solely focused on the experiences of disability service providers.

### Preparedness

Joint working appeared to be influenced by the level of preparedness in acute services to provide healthcare for people with intellectual disabilities. Preparedness was interlinked with the knowledge, skills, attitudes, and commitment possessed by staff in acute services. When prepared, staff in acute services had a better understanding of the needs of people with intellectual disabilities, which promoted joint working. The converse was also found where, at times, acute services were unprepared to provide care to this group.

Staff in acute services recalled feeling nervous and uneasy when persons with intellectual disabilities entered their care because they didn’t know what to expect and felt unprepared to meet the needs of the person (Brown and Guvenir, 2008). One staff nurse described feeling *“thrown in at the deep end”* (pg 112) and felt that the acute service was better prepared to care for the person when admission was planned (Brown and Guvenir, 2008)*.* Identifying those with intellectual disabilities as they enter acute services was viewed as a pre-requisite to preparing acute service staff to provide appropriate healthcare and put necessary adjustments in place (Oulton et al., 2018; Oulton et al., 2019). Not being fully prepared resulted in staff in acute services relying on carers to provide direct care to the individual (Brown and Guvenir, 2008; Drodz and Clinch, 2016). In addition, a deficit in knowledge on the needs of this population group and services available to this cohort in the community was identified as contributing to unpreparedness and prevented joint working. Appropriate training in intellectual disability, community supports, communication strategies and positive behaviour support are required to prepare staff in acute services to support people with intellectual disabilities (Oulton et al., 2015; Oulton et al., 2019).

On the contrary, there was also evidence of acute services being prepared to meet the needs of people with intellectual disabilities. Staff in acute services recognised that more work is needed to improve healthcare for persons with intellectual disabilities and demonstrated a willingness to get it right (Oulton et al., 2019). Drodz and Clinch (2016) identified that nurses possess the same level of compassion for those with intellectual disabilities as those without. Nurses had the same level of courage to speak up and advocate for patients with intellectual disabilities as they would for other patients. Importantly, staff in acute services expressed a strong commitment to provide quality healthcare (Drodz and Clinch, 2016).

Although, suboptimal preparedness exists there are clear indications that acute services are prepared for change. This change can be influenced by the commitment demonstrated by hospital staff to improve healthcare for persons with intellectual disabilities.

### Theme 1: Responsibility to improve healthcare

This theme captures the responsibilities of both acute and disability service providers in improving healthcare for persons with intellectual disabilities within acute services. It is suggested that some aspects of healthcare for persons with intellectual disabilities in acute services are poorer than for those without (Drodz and Clinch, 2016; Oulton et al., 2019). Despite this, there was a clear indication that acute service providers were prepared for and attempting change to improve this populations healthcare experiences.

Specific intellectual disability policies and protocols support and prepare staff in acute services to provide appropriate healthcare to those entering the service (Bell, 2012) and are viewed as a prompt for acute service staff to think about the person’s needs (Oulton et al. 2018). Bell (2012) emphasized the need for hospitals to develop specific intellectual disability policies, in this case to support the implementation of hospital passports. Despite this, the absence of appropriate specific intellectual disability policies and protocols, was evident in some studies (Bell, 2012; Oulton et al., 2018). In one instance, when examining the organisational perspective on the delivery of healthcare for children with intellectual disabilities across 22 acute service sites, Oulton et al. (2018) discovered no standalone intellectual disability policy existed. Instead, care of persons with intellectual disabilities was incorporated into mainstream policies but could not always be referred to as they were unsuitable in meeting their specific needs.

The absence of specific intellectual disability polices and protocols in acute services is intrinsically linked to confusion around who is responsible for their development and implementation. Tuffrey-Wijne et al. (2014) found that a lack of clarity around who is responsible for implementing reasonable adjustments prevented continuity of care. Furthermore, healthcare professionals in acute services outlined an absence of organisational and managerial commitment to certain aspects of healthcare for persons with intellectual disabilities such as the implementation of hospital passports (Oulton et al. 2018) and recruitment of intellectual disability liaison nurses. Intellectual disability liaison nurses were regarded as a valuable source of expertise to improve healthcare for persons with intellectual disabilities. They play a role co-ordinating care (MacArthur et al., 2015; Brown et al., 2012), collaborating with other professionals in the community (Oulton et al., 2019) and therefore bridge the gap between services (Castles, 2012). Intellectual disability liaison nurses in acute services prepare staff in acute services to provide care to those with intellectual disabilities (MacArthur et al. 2015) by providing training and education on areas such as communication, consent, and the health needs of people with an intellectual disability (Brown et al., 2012). Despite their importance in acute services, intellectual disability liaison nurses were not available at the weekends, or after hours (Brown et al., 2012) and there was a need for increased visibility of the role in acute services (Castles, 2012). It was recognised that organisational and managerial support is required to enhance the role of the intellectual disability liaison nurse in acute services (Tuffrey-Wijne et al., 2014).

Despite evidence identifying a lack of specific intellectual disability policies and protocols in acute services and confusion around roles and responsibility between services, there is evidence which indicates positive efforts are being made to improve healthcare experiences of persons with intellectual disabilities. As acute service providers commit to the delivery of appropriate healthcare to patients with intellectual disabilities, positive practices are becoming embedded over time (Oulton et al., 2019). Current evidence recognises the need for reasonable adjustments such as specific intellectual disability policies and protocols, communication tools and introduction of intellectual disability liaison nurses across acute services. However, these cannot be achieved without commitment and responsibility from acute services. Although acute services are demonstrating positive signs of progress in delivering quality healthcare to this group, progress will be slowed if responsibility to drive change is not established.

### Theme 2: Information-sharing between service providers

The importance of, and need for, effective communication and information-sharing between acute and disability service providers to improve healthcare experiences of people with an intellectual disability was a recurring theme across seven studies (Brown & Guvenir, 2008; Lewis & Stenfert-Kroese, 2010; Bell, 2012; Tuffrey-Wijne et al., 2014; Oulton et al., 2015; Drodz & Clinch, 2016; Oulton et al., 2018; Spassiani et al., 2020). Oulton et al. (2015) claims that more information-sharing is required between all agencies involved to improve healthcare experiences for this group.

Poor communication between services was repeatedly recognized despite measures being in place to facilitate the process (Bell, 2012; Drodz and Clinch, 2016; Oulton et al., 2018). There was a lack of appropriate and standardised flagging and identifying systems in place within hospitals to alert hospital staff to persons with an intellectual disability entering their care (Tuffrey-Wijne et al., 2014, Oulton et al., 2015; Oulton et al., 2018; Oulton et al., 2019; Spassiani et al., 2020). Staff in acute services detailed situations where a lack of forewarning impacted the care they provided and resulted in unsuccessful visits (Tuffrey-Wijne et al. 2014). In most cases identifying persons with intellectual disabilities took place when the individual had already presented to the hospital, and the persons intellectual disability diagnosis was shared between colleagues or by the carer accompanying the individual (Tuffey-Wijne et al., 2014). Having appropriate flagging systems in place enables hospitals to put appropriate reasonable adjustments in place in a timely manner, rather than as the person advances through their healthcare journey (Oulton et al., 2019).

The use of hospital passports was another measure repeatedly recognised to promote information-sharing between both services (Bell, 2012; Drodz and Clinch, 2016; Oulton et al., 2018). Hospital passports are a valuable source of information and are typically completed in conjunction with the person themselves, carers and health and social care staff (Bell, 2012). They are viewed as a link between hospital and community and support continuity and communication of care between both services (Bell, 2012). The implementation of hospital passports support staff in acute services to gain knowledge and a better understanding of the patient in their care (Oulton et al., 2018). However, it has been noted that the document is not always used by hospital staff (Tuffrey-Wijne et al., 2014) as staff in acute services reported being too busy to read it (Bell, 2012). Furthermore, when examining the organisational context for healthcare delivery for children and young persons with intellectual disabilities, Oulton et al. (2018) found that in some cases, hospital passports were not always in place and a lack of organisational commitment hindered implementation. In a study exploring healthcare professionals’ experiences of using the document (Bell, 2012), a healthcare worker recalled a positive experience where the hospital passport was passed to all relevant healthcare professionals in the acute service which resulted in staff in acute services being informed of the persons likes, dislikes and communication needs thus highlighting its value when implemented appropriately.

The sharing of information was also facilitated via the paid carer accompanying the individual to hospital. Carers are viewed as invaluable in providing important information to hospital staff (Castles, 2012). Some nursing staff report challenges in meeting the needs of patient with intellectual disabilities without carers present (Brown and Guvenir, 2008). If healthcare professionals requested for carers to leave after visiting hours it was viewed as poor reasonably adjusted healthcare (Tuffrey-Wijne et al., 2014). Albeit in some cases, carers were not always familiar with the individual they were supporting in hospital (Bell., 2012) thus impacting information-sharing and the potential for joint working between services. Overall, the evidence highlights a continuing over-reliance on carers to remain in hospital and provide direct care to the person in lieu of acute service staff (Lewis and Stenfert-Kroese, 2010; Drodz and Clinch, 2016).

The current evidence indicates a need for robust information-sharing between both services. Several measures are in place to facilitate the process of information-sharing however the evidence suggests they are not always used to their full potential.

### Theme 3: Knowledge, awareness, and training

Limited knowledge and awareness of the needs of people with intellectual disability amongst healthcare professionals in acute services was evident across several studies (Tuffrey-Wijne et al., 2014; Oulton et al., 2015; Drodz and Clinch, 2016; Oulton et al. 2018; Spassiani et al., 2020). and captured a recurring trend of knowledge and training gaps impacting healthcare delivered to those with intellectual disabilities.

When examining the knowledge and skills of emergency department nurses when caring for patients with intellectual disabilities, nurses self-reported having limited knowledge of intellectual disability (Spassiani et al., 2020) in addition to medical, dental staff and healthcare assistants (Tuffrey-Wijne et al., 2014). Inadequate knowledge and awareness of communication and positive behaviour supports contributed to reduced confidence and competence for caring for patients with an intellectual disability in acute services (Lewis and Stenfert-Kroese, 2010; Bell, 2012; Drodz and Clinch, 2016; Oulton et al., 2018). Participants reported feelings of anxiety and fear when providing healthcare to persons with intellectual disabilities, particularly in cases of emergency admission or if persons presented with behaviour that challenge (Brown and Guvenir, 2008; Oulton et al., 2015). Feelings of fear led healthcare professionals to become hesitant when providing direct care to individuals (Lewis and Stenfort-Kroese, 2010).

Furthermore, Spassiani et al., (2020) identified that staff within acute services had limited knowledge and awareness of services and resources available for persons with intellectual disabilities in the community. For those who had awareness of community services and resources, staff expressed uncertainty about how they could access these services if required when providing care to those with an intellectual disability. Oulton et al. (2018) concludes that specific intellectual disability training provides all staff in acute services with the knowledge to access appropriate specialist staff and resources when needed.

The decreased knowledge and awareness of the needs of people with intellectual disabilities was linked to a deficiency in the availability of appropriate training (Oulton et al., 2019). Failure to provide specific training to equip staff in acute services to care for persons with intellectual disabilities results in a limited understanding of their needs (Tuffrey-Wijne et al., 2014; Oulton et al., 2015). Staff in acute services reported that the knowledge they possessed came from past experiences rather than specific training provided and indicated they need further training in communication strategies, positive behaviour support (Oulton et al., 2015), and awareness of reasonable adjustments (Tuffrey-Wijne et al., 2014)

It is clear from the current evidence that staff in acute services themselves identify a gap in their knowledge and would benefit from further training in communication, behaviour support, reasonable adjustments to better enable them to improve their confidence and provide optimum healthcare to this group.

## Discussion

The healthcare of persons with intellectual disabilities in acute services has been the focus of several reports and inquiries over the last decade (Mencap, 2007, Michael 2008; Heslop et al., 2013; Heslop et al., 2021). Following these reports and inquiries, much research has centred around gaining a deeper understanding of the experiences of this population group while accessing acute care services (Tuffrey-Wijne et al., 2014; Tuffrey-Wijne & Hollins, 2014; Walker et al., 2022; Howieson, 2015; Philips, 2019; McCormick et al., 2021). Research has sought to identify methods to overcome the issues they encounter in acute services. Joint working is repeatedly recognised as one way of achieving this (Lewis and Stenfert-Kroese, 2010; Cantrell et al., 2020; Moloney et al., 2021). Joint working between health and social care services is key to improving the health of the population as collaboration between healthcare professionals across services enables the sharing of their knowledge, skills, and expertise to benefit service users (Alderwick et al., 2021). Despite this, there appears to be a paucity of research specifically exploring joint working between acute and disability service providers.

All twelve studies centred around acute services and gathered the views of healthcare professionals working within this setting who provided healthcare to persons with intellectual disabilities and only six studies included the views of healthcare professionals within services in the community (Brown and Guvneir, 2008; Brown et al., 2012; Bell, 2012; Castles, 2012; Tuffrey-Wijne et al., 2014; MacArthur et al., 2015). No identified study specifically set out to explore joint working between disability and acute service providers, instead references to joint working and/or non-joint working between both services was alluded to in study findings. However, at times it was difficult to ascertain whether joint working/non-joint working between the services took place. In studies where this was referred to, the findings failed to detail how joint working/non-joint working contributed to the healthcare provided to people with intellectual disabilities in acute services.

The findings of this MMSR highlight the need for more effective joint working between both services. Improving joint working requires services to better understand its process. The process of joint working between services begins, firstly with a mutual understanding of the benefits of working together (Cameron et al., 2013) and secondly an understanding of who is responsible for the process (Health Service Executive, 2017). A degree of uncertainty around who is responsible for the development of specific intellectual disability policies and protocols was found to inhibit the implementation of reasonably adjusted healthcare. This is in keeping with a study by Brown et al. (2016) who found that failure to recognise roles and responsibilities, negatively impacts the delivery of appropriate person-centred healthcare. In a joint working protocol between primary care, disability and child and adolescent mental health services in Ireland, the Health Service Executive (2017) outlined that services must mutually decide upon their roles and responsibilities and agree which service is to take the lead to co-ordinate healthcare.

There was an emphasis on the need for specific intellectual disability policies and protocols to improve outcomes for persons with intellectual disabilities in acute services (Bell., 2012; Oulton et al., 2018). Brown & MacArthur (2006) identified a need for policies and protocols to be developed and shared across health and social services to improve hospital outcomes for people with intellectual disabilities. Having specific polices and pathways in place affords staff the opportunity to consider the needs of the patient and what may be required to support the person on their healthcare journey (Oulton et al., 2018). The presence of policies and protocols in healthcare ensure that services are compliant with regulatory and legal obligations. Policies influence decision-making therefore their importance in healthcare cannot be underestimated (O Donnell & Vogenberg, 2012). Despite agreement that specific intellectual disability policies and protocols are required in acute services (Bell., 2012; Oulton et al., 2018) they are inconsistently present across services. Some acute services incorporated the care of persons with intellectual disabilities into mainstream policies and is viewed as a means of promoting inclusion, which has been a priority for modern-day policymakers (Bigby, 2020). While the benefit of having specific policies relating to intellectual disability are noted, it can be argued that developing such policies may result in the stigmatisation of those with intellectual disabilities (Bigby, 2020).

The success of joint working between both disability and acute service providers appeared to be influenced by inter-service communication. The evidence indicates inadequate communication existed between services even when, at times, measures are in place to support the process (Bell, 2012; Drodz and Clinch, 2016; Oulton et al., 2018). This finding is in keeping with those of two previous systematic reviews (Iacono et al. 2014; McCormick et al. 2021) which recognised the impact of poor communication on the experiences of those with intellectual disabilities in acute services. Without effective communication between all stakeholders in healthcare, patient safety and continuity of care become compromised (Vermeir et al., 2015). The findings indicate a need for increased information-sharing between services to improve outcomes for people with intellectual disabilities, but this can only be achieved with improved communication between services (Oulton et al. 2015). Information-sharing is key to facilitating the exchange of expert knowledge and supports collaborative, integrated and continuous care (Kariotis et al., 2019) therefore improving the efficiency and performance of services involved (Yang & Maxwell., 2011).

There was agreement amongst healthcare professionals that the hospital passport is a beneficial tool to support communication and information-sharing, but they are under-valued and under-utilized within acute services (Drodz and Clinch, 2016). The challenges to utilization of the document have been attributed to the variation in the format of the hospital passport across different services, missing key information, poor accessibility, and a lack of commitment from acute services to use the tool (Northway et al., 2017; Oulton et al., 2018). Recommendations have been made for a standardised approach to the hospital passport format to improve the uptake and utilization of the document within acute services (Northway et al.,2017, Lunksy, 2018), but this can only be achieved by individuals, carers and service providers working together (McCormick et al., 2021). The future success of the hospital passport to support joint working is reliant on acceptance and commitment from healthcare professionals and require a strong level of managerial commitment, policy engagement, and consultation with all those involved going forward (Leavey et al. 2020).

Challenges in identifying and flagging patients with intellectual disabilities in acute services were also identified (Tuffrey-Wijne et al., 2014; Oulton et al., 2018; Oulton et al., 2019). Flagging systems are a mechanism of recognising vulnerable persons like those with intellectual disabilities to facilitate the timely implementation of reasonable adjustments to improve hospital experiences (Ong et al., 2022). While the benefits of identifying this group using flagging systems in acute services have previously been documented (Tuffrey-Wijne & Hollins, 2014; Walker et al., 2022), concerns have been raised about the potential labelling of this group (Kenten et al., 2019). Healthcare professionals in acute services instead suggest that flagging systems would be more beneficial if they contained information about what the diagnosis means for the person and the reasonable adjustments required to support them in hospital rather than just flagging the diagnosis (Oulton et al. 2015).

In line with past research, it was found that healthcare professionals in acute services possess limited knowledge and awareness in the field of intellectual disability which resulted in reduced confidence in caring for this group (Salvador-Carulla & Saxena, 2009; Appelgren et al., 2018; Ong et al., 2022). Not having the knowledge and skills to meet their needs at times resulted in negative attitudes and emotions towards individuals with intellectual disabilities (Desroches et al., 2021). While these feelings and attitudes are not intentional, they are avoidable through the delivery of specific training and education. It is crucial for healthcare professionals to receive specific training in intellectual disability to increase their knowledge and understanding of this group, but importantly they must have the skills to transfer it to their practice (Oulton et al., 2019). The deficit in knowledge contributes to an over-reliance on carers to provide direct care to the individual they support while in hospital (Iacono et al., 2014). While having carers present is reassuring for hospital staff (Hemsley, Balandin, & Worrall, 2011), it may not always feasible for disability services to provide carers to support the person during their hospital stay. Providing formal training on the needs of people with intellectual disability will equip staff with the appropriate knowledge to provide optimum healthcare to those with an intellectual disability and reduce dependency on carers.

While majority of the findings in this review imply an absence of joint working between both services, there is encouraging evidence which demonstrates both a willingness and commitment to improve healthcare and hospital experiences and for people with intellectual disabilities. It is important to acknowledge the positive attitudes of healthcare professionals in acute services towards people with an intellectual disability and the desire to deliver optimum healthcare to this group. At an organizational level, acute services have made some positive advancements towards improving their service for those with intellectual disabilities which was evidenced in the establishment of intellectual disability liaison nurse posts. They contribute to better care for persons with intellectual disabilities (Castles, 2012; McCarron et al., 2018) as they are responsible for co-ordinating healthcare and are viewed as a means of bridging the gap between both services (Brown et al., 2012; MacArthur et al., 2015). Intellectual disability liaison nurses are valuable assets within acute services as they play vital roles in training and educating staff, implementing reasonable adjustments, identifying persons with intellectual disabilities, supporting information-sharing, and creating and implementing policies and pathways (Castles, 2012; MacArthur et al., 2015; Drodz & Clinch, 2016; Oulton et al., 2019). However, they are not always accessible after hours or at weekends (Drodz & Clinch, 2016) therefore impacting on the opportunity for joint working between acute and disability service providers.

## Strengths and limitations

A particular strength to this study was the inclusion of studies from 2006 onwards. Including studies from this year onwards captured studies conducted post the enactment of the United Nation Convention on the Right of Persons with Disabilities (United Nations, 2006), and at a time when the rights of people with disabilities were placed at the centre of changes to legislation and policy.

While this was a comprehensive review of the current body of literature on joint working between acute and disability service providers, the study is not without its limitations. An aim of this review was to explore disability service providers experiences of joint working/ non-joint working with acute service providers. The review revealed limited research on disability service providers view of joint working and so the review failed to address this research question. Only studies conducted in English were included in the review. Perhaps including studies in other languages may have expanded the scope of this review and revealed data relating to joint working between acute and disability service providers.

## Conclusion

Past research has explored the healthcare experiences of those with intellectual disabilities while accessing healthcare in acute services as well as identifying measures to overcome such challenges. Joint working between service providers has been recognised to improve healthcare in acute services for this group, as it is inevitable that those with an intellectual disability will continue to require access to both acute and disability services into the future.

There is a need for increased joint working between both services in practice. The success of joint working is dependent on information-sharing, and information sharing is dependant on communication. Adopting open channels of communication will assist services in overcoming challenges and promote joint working going forward. Intellectual disability liaison nurses are necessary to support joint working and bridge the gap between services, however there is a recognised need for increased commitment from acute services to ensure the effectiveness of the role.

Providing training and education for staff in acute services is required to better equip them to provide healthcare for people with intellectual disabilities. The review discovered a gap in knowledge in acute services on the awareness on the services available to people with intellectual disabilities in the community. Having knowledge of these services, will enable staff to reach out to these services and therefore increase the prospect of joint working.

The findings recognise a need for the development of specific intellectual disability policies and protocols. With that said, it is unclear what aspect of care needs to be incorporated into these specific documents that cannot be addressed within mainstream policies and if their presence could support joint working between both services. While the evidence offers acute service providers views on the need for such documents, there appears to be a gap in relation to intellectual disability service providers views on policies and protocols. In Ireland there is a joint working protocol in place between primary care, disability, and mental health services which supports a joint working relationship between the services. This should be investigated further to establish if the same principles can be transferred and applied to acute and intellectual disability services and if the presence of a protocol can increase joint working going forward. The development of policies and protocols to support joint working would benefit from the input of both services to ensure its success.

The current evidence captures the experiences of acute service providers but there appears to be a dearth of information available on the experiences of disability service providers of joint working and/or non-joint working with acute service providers. Further research to explore the views of disability service providers is required to add to the findings of this review to provide a better understanding of what is working well, not working well and what is required to support joint working between both services going forward.
